# Heterologous biosynthesis of isobavachalcone in tobacco based on *in planta* screening of prenyltransferases

**DOI:** 10.3389/fpls.2022.1034625

**Published:** 2022-10-06

**Authors:** Lirong Guo, Wei Zhao, Yan Wang, Yu Yang, Cuimei Wei, Jian Guo, Jianye Dai, Masami Yokota Hirai, Aike Bao, Zhigang Yang, Haijuan Chen, Yimeng Li

**Affiliations:** ^1^ School of Pharmacy, Lanzhou University, Lanzhou, China; ^2^ RIKEN Center for Sustainable Resource Science, Yokohama, Japan; ^3^ College of Pastoral Agriculture Science and Technology, Lanzhou University, Lanzhou, China; ^4^ Key Laboratory of Medicinal Animal and Plant Resources of Qinghai-Tibetan Plateau, Academy of Plateau Science and Sustainability, Qinghai Normal University, Xining, China

**Keywords:** isobavachalcone, prenyltransferase, prenylflavonoids, *de novo* biosynthesis, multigene expression vector, plant metabolic engineering

## Abstract

Isobavachalcone (IBC) is a prenylated chalcone mainly distributed in some Fabaceae and Moraceae species. IBC exhibits a wide range of pharmacological properties, including anti-bacterial, anti-viral, anti-inflammatory, and anti-cancer activities. In this study, we attempted to construct the heterologous biosynthesis pathway of IBC in tobacco (*Nicotiana tabacum*). Four previously reported prenyltransferases, including *GuILDT* from *Glycyrrhiza uralensis*, *HlPT1* from *Humulus lupulus*, and *SfILDT* and *SfFPT* from *Sophora flavescens*, were subjected to an *in planta* screening to verify their activities for the biosynthesis of IBC, by using tobacco transient expression with exogenous isoliquiritigenin as the substrate. Only SfFPT and HlPT1 could convert isoliquiritigenin to IBC, and the activity of SfFPT was higher than that of HlPT1. By co-expression of *GmCHS8* and *GmCHR5* from *Glycine max*, endogenous isoliquiritigenin was generated in tobacco leaves (21.0 μg/g dry weight). After transformation with a multigene vector carrying *GmCHS8*, *GmCHR5*, and *SfFPT*, *de novo* biosynthesis of IBC was achieved in transgenic tobacco T_0_ lines, in which the highest amount of IBC was 0.56 μg/g dry weight. The yield of IBC in transgenic plants was nearly equal to that in *SfFPT* transient expression experiments, in which substrate supplement was sufficient, indicating that low IBC yield was not attributed to the substrate supplement. Our research provided a prospect to produce valuable prenylflavonoids using plant-based metabolic engineering.

## Introduction

Prenylflavonoids are a subclass of naturally occurring flavonoids that contain at least one lipophilic prenylated side-chain in the flavonoid skeleton. They are mostly found in a small number of families, including Fabaceae, Moraceae, Cannabaceae, Clusiaceae, Apiaceae, and Euphorbiaceae (Yang et al., 2015). Prenylflavonoids act as phytoalexins and defend plants against infections through their antibacterial activities (Ahuja et al., 2012). They are also bioactive substances that have a variety of biological effects, such as estrogenic (An et al., 2016; Stulikova et al., 2018), antioxidant (Botta et al., 2005b), anti-cancer (Saito et al., 2018; Sastre-Serra et al., 2019), and antiviral (Grienke et al., 2016). The addition of various prenyl groups to the aromatic ring of flavonoids not only gives rise to the structural diversity, but also significantly increases their biological activity and bioavailability in comparison to their non-prenyl flavonoid parent compounds. Such enhancement may be caused by the presence of a lipophilic prenyl side-chain (Zhao et al., 2003; Kretzschmar et al., 2010). It is thought that the prenyl side-chain improves the membrane permeability of prenylated flavonoids, which subsequently provides enhanced biological activities (Botta et al., 2005a; Mukai, 2018; Levisson et al., 2019).

Prenylflavonoids often exist at trace levels and are distributed in limited species (Yang et al., 2015; Gomes et al., 2022). Isobavachalcone (IBC), a prenylated chalcone at the 3’ position, is mainly isolated from several species belonging to the Fabaceae and Moraceae including the traditional Chinese medicinal materials *Psoralea corylifolia* as one of the major active components (Chen et al., 2012; Gao et al., 2016; Xing et al., 2022). According to reports, this prenylated chalcone exhibits a wide range of physiological and pharmacological properties, including antioxidant, anti-inflammatory, anti-rheumatoid arthritis, anticancer, attenuate hepatocyte injury, and neuroprotective properties (Dzoyem et al., 2017; Li et al., 2019a; Zhou et al., 2019; Song et al., 2021; Wang et al., 2022). In *P. corylifolia*, IBC is only distributed in its seeds at low concentration (Tsai et al., 1996). Due to regioselectivity, stereoselectivity, and laborious chemical reaction steps, the chemical synthesis of prenylflavonoids is subjected to various restrictions, which have hampered its production at scale and commercial application (Gester et al., 2001; Tischer and Metz, 2007; Nemoto et al., 2012; Grealis et al., 2013).

**Figure 1:**
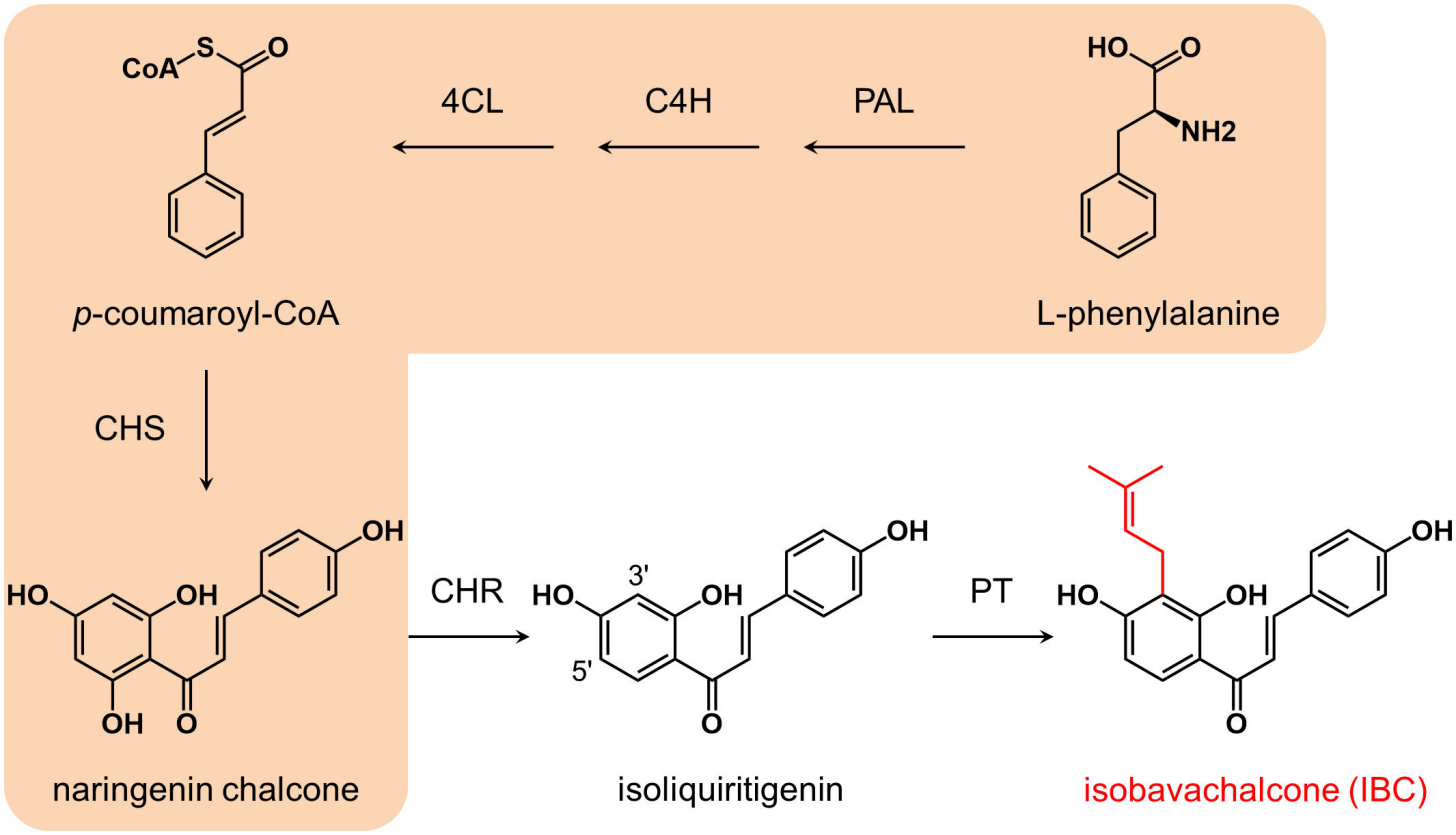
Biosynthesis pathway of isobavachalcone. Enzymes and compounds in the orange shadow commonly exist in higher plants, while CHR is mainly distributed in leguminous plants. Isobavachalcone is supposed to be synthesized *via* 3’ prenylation of isoliquiritigenin by a PT. PAL, phenylalanine ammonia-lyase; C4H, cinnamic acid 4-hydroxylase; 4CL, 4-coumarate: coenzyme A ligase; CHS, chalcone synthase; CHR, chalcone reductase; PT, prenyltransferase.

Based on the structure, isoliquiritigenin is thought to be the biosynthetic precursor of IBC (Li et al., 2018). The biosynthesis of isoliquiritigenin is derived from the phenylpropanoid pathway (**Figure 1**). Phenylalanine is converted to naringenin chalcone (2’,4,4’,6’-tetrahydroxychalcone) by the successive catalysis of phenylalanine ammonia-lyase (PAL), cinnamate 4-hydroxylase (C4H), 4-coumarate: coenzyme A ligase (4CL), and chalcone synthase (CHS) (Yi et al., 2010). Naringenin chalcone is then isomerized to naringin either spontaneously or *via* chalcone isomerase (CHI) (Ralston et al., 2005). In the Fabaceae family, CHS-catalyzed condensation reaction is coupled with chalcone reductase (CHR)-catalyzed reduction reaction to generate isoliquiritigenin (2’,4,4’-trihydroxychalcone) (Mameda et al., 2018). Although no specific prenyltransferase catalyzing the biosynthesis of IBC has been characterized from *P. corylifolia*, several chalcone- and flavanone-specific prenyltransferases have been identified from some legume and nonlegume species. CtIDT from *Cudriana tricuspidata*, MaIDT from *Morus alba* (Wang et al., 2014), GuILDT from *Glycyrrhiza uralensis* (Li et al., 2018), and SfILDT from *Sophora flavescens* (Sasaki et al., 2011) can recognize isoliquiritigenin as a substrate; CtIDT, MaIDT, and GuILDT catalysis the prenylation of isoliquiritigenin at C-3’ position to generate IBC, while SfILDT prenylates isoliquiritigenin at C-5’ position. On the other hand, HlPT1 from *Humulus lupulus* recognizes naringenin chalcone as a substrate to form desmethylxanthohumol (Tsurumaru et al., 2012). In addition, SfFPT (Chen et al., 2013) and SfN8DT (Sasaki et al., 2008) from *S. flavescens* exhibit substrate specificity towards liquiritigenin, which is the isomerization product of isoliquiritigenin.

The enzymatic synthesis of IBC was achieved by *in vitro* catalysis by GuILDT (Li et al., 2018). Recently, heterologous biosynthesis of isoliquiritigenin in *Saccharomyces cerevisiae* and *Yarrowia lipolytica* with high amount was reported (Yin et al., 2020; Akram et al., 2021). As an alternative, *Nicotiana* spp. have been widely used as the hosts in plant-based metabolic engineering to produce a range of natural compounds, including astaxanthin (Mann et al., 2000; Zhu et al., 2007; Hasunuma et al., 2008; Lu et al., 2017; Mortimer et al., 2017; Allen et al., 2022), crocin (Martí et al., 2020; Ahrazem et al., 2022), colchicine (Nett et al., 2020), taxadiene-5α-ol (Li et al., 2019b), deoxypodophyllotoxin and its derivatives (Lau and Sattely, 2015; Schultz et al., 2019), and isoflavonoids (Yu et al., 2000; Tian and Dixon, 2006; Liu et al., 2007; Pandey et al., 2014). CHR or flavonoid prenyltransferases do not exist in *Nicotiana* spp. (Sierro et al., 2014). However, naringenin chalcone, the precursor of IBC, is derived from the phenylpropanoid pathway, which is conserved in higher plants. Moreover, when constructing a chalcone/flavonoid pathway, the endogenous phenylpropanoid pathway in *Nicotiana* hosts can be utilized to reduce the target genes to be transformed. In this study, to attempt the heterologous biosynthesis of IBC in tobacco (*Nicotiana tabacum*), we selected four prenyltransferases (SfFPT, SfILDT, GuILDT, and HlPT1) as the candidates to construct the biosynthesis pathway of IBC based on the similarity of their *in vitro* substrate structures (isoliquiritigenin, naringenin chalcone, and naringenin) (**Supplementary Figure 1**). We conducted an *in planta* screening of these prenyltransferases using tobacco transient expression system, and found that SfFPT and HlPT1 could convert exogenous isoliquiritigenin to IBC. It is unfeasible to supply exogenous substrates when plants are applied for the large-scale production of natural compounds under field conditions. To achieve the heterologous biosynthesis of IBC in tobacco, endogenous biosynthesis of isoliquiritigenin is necessary. The presence of isoliquiritigenin is resulted from the combined enzymatic activities of CHS and CHR. By co-expression of *GmCHS8* and *GmCHR5* (Mameda et al., 2018), we synthesized isoliquiritigenin in tobacco as the endogenous substrate. Finally, the *de novo* biosynthesis of IBC was achieved in tobacco (0.56 μg/g DW) by introducing a multigene expression vector containing *GmCHS8*, *GmCHR5*, and *SfFPT* genes. Our study provided a potential source of this valuable natural compound.

## Materials and methods

### Plant materials and chemicals

After sterilization with 70% ethanol, tobacco (*Nicotiana tabacum*) seeds were sown on soil made up of a 3:1:1 combination of peat moss, perlite, and vermiculite (v/v/v). A fluorescent light cycle of 16/8 hours and a temperature of 26°C were used to cultivate seedlings. Seeds of soybean (*Glycine max*), *Sophora flavescens*, *Glycyrrhiza uralensis*, and *Humulus lupulus* were cultivated under the same condition. The standard compounds isobavachalcone (IBC) (CAS: 20784-50-3) and isoliquiritigenin (CAS: 961-29-5) were purchased from ShanghaiyuanyeBio-Technology Co., Ltd. (Shanghai, China).

### Nucleic acid extraction and cDNA synthesis

Six-week-old plants of soybean, *S. flavescens*, *G. uralensis*, and *H. lupulus* were treated with 1% yeast extract for 24 h. Roots and leaves were collected in 2 mL tubes after being frozen in liquid nitrogen, and stored at -80°C until use. A 5 mm glass bead was placed within each tube. An automated grinder (Jingxin, Shanghai, China) was used to homogenize the liquid nitrogen cooling samples. Total RNA was isolated from samples less than 80 mg by using an EasyPure Plant RNA Kit (TransGen Biotech, Beijing, China) following the standard protocol. cDNA was synthesized from total RNA using a RiverTra Ace qPCR RT Master Mix and the gDNA Remover Kit (Toyobo, Tokyo, Japan). cDNA was diluted to 1/10 with nucleic acid-free water.

### Gene cloning and plasmid construction

cDNA isolated from the soybean, *S. flavescens.*, *G. uralensis*. and *H. lupulus*. was used as templates for the cloning of genes. The coding sequence of *GmCHS8* (AY237728), *GmCHR5* (LC309095), *SfFPT* (KC513505), *SfILDT* (AB604223), *GuILDT* (KR139751), and *HlPT1* (AB543053) were amplified using a KOD plus DNA polymerase (Toyobo, Tokyo, Japan). *attB1* and *attB2* sequence was attached to 5’ sites of forward and reverse primers, respectively. The sequence of primers was listed in **Supplementary Table 1**. Amplified products were analyzed on a 1.2% agarose gel with 1×TAE and run at 100 V to confirmed their size. The gel was stained with GelGreen (Biotium, CA, USA), and the bands were cut out under blue light. The bands were purified with an AxyPrep DNA Gel Extraction Kit (Axygen, CA, USA). Each gene was introduced into a pDONR222 vector by the Gateway BP reaction (Invitrogen, CA, USA). The sequence was confirmed by sequencing using a M13 forward primer. The sequence of all genes was the same as the reference from GenBank. The target genes were introduced into a pGWB402Ω vector (Nakagawa et al., 2007) by the Gateway LR reaction (Invitrogen, CA, USA), generating six single gene expression vectors, i.e., pGWB402Ω-GmCHS8, pGWB402Ω-GmCHR5, pGWB402Ω-SfFPT, pGWB402Ω-SfILDT, pGWB402Ω-GuILDT, and pGWB402Ω-HlPT1, in which each CDS was flanked with an enhanced cauliflower mosaic virus (CaMV) 35S promoter and a *NOS* terminator.

The multigene expression vector was constructed based on the Golden Gate assembly strategy (Engler et al., 2008; Bell and Molloy, 2022) using a T4 DNA Ligase (New England Biolabs, CA, USA) and the restriction enzyme BsaI-HF v2 (New England Biolabs, CA, USA). A recognition site for the *BsaI* restriction enzyme on *GmCHS8* was changed by point mutation using PCR. Primers for mutation were designed based on the degenerate codons. The PCR amplified products were subsequently ligated using a pEASY-Basic Seamless Cloning and Assembly Kit (TransGen Biotech, Beijing, China), and then subcloned into a pGWB402Ω vector. The cassettes spanning the promoter-CDS-terminator regions of each gene in pGWB402Ω vector were amplified using primers attached with *BsaI* restriction enzyme and overhanging sequences, which were designed by Golden Gate Assembly Tool (https://goldengate.neb.com/). All cassettes were assembled into an pPZP vector backbone (Hajdukiewicz et al., 1994). This vector was named pGK-IBC, which contained a *nptII* gene conferring kanamycin resistance plants, an *Sm^R^* gene conferring spectinomycin resistance to bacteria, and *Ori* and *OriV* replicons. In addition, the coding sequence of *AtMYB28* (NM_125535) from *Arabidopsis thaliana* was cloned using primers flanked with *KpnI* restriction enzyme sites. The amplicon was subcloned into the pGWB402Ω vector, and *AtMYB28* was removed by enzymatic cleavage to generate an empty vector.

### Transient expression

The single gene expression vector pGWB402Ω-SfFPT, pGWB402Ω-SfILDT, pGWB402Ω-GuILDT, and pGWB402Ω-HlPT1, as well as multigene expression vector pGK-IBC were transformed into *Agrobacterium tumefaciens* strain GV3101. After validation of transformation by colony PCR, the bacteria were grown in a liquid LB medium for 24 hours at 28°C. The suspension was discarded after the bacteria were separated by centrifugation for 10 min at 4000 g at 4°C. The pellets were gently resuspended to an OD600 of 1.5 in sterilized water containing 0.2 mM acetosyringone, 10 mM MgCl_2_, and 10 mM MES (pH = 5.8). The suspension was shaken at 120 rpm for two hours in dark at room temperature. Tobacco leaves that were six weeks old and fully expanded were employed for agroinfiltration. Using a 1 ml needleless syringe, *Agrobacterium* suspension was injected into the whole leaf area through stomas from the abaxial side of the leaves. Plants were cultivated after agroinfiltration for 24 hours in the dark, and then transferred to the normal condition. For isoliquiritigenin supplement, agroinfiltrated tobacco leaves were injected with 100 μM isoliquiritigenin five days after agroinfiltration. After cultivation under normal conditions for 24 h, injected leaves were sampled. Tobacco leaves infected with *Agrobacterium* carrying an empty vector served as a negative control.

### Plant transformation

Genetic transformation of tobacco was performed based on previous protocols (Horsch et al., 1985; Rogers et al., 1986). Leaf explants from healthy tobacco were soaked in 30% commercial bleach for 10 minutes on a rotating shaker at 100 rpm at room temperature, and washed three times with sterilized ultrapure water. *A. tumefaciens* containing the pGK-IBC or empty vectors was collected by centrifugation for 10 min at 4000 g at 4°C, and resuspended in liquid MS0 medium to an OD600 of 0.4. About 1 cm^2^ explants were infected with the *A. tumefaciens* suspension for 5 minutes. The remaining bacterial solution on the explant surface of the material was absorbed using sterile filter paper. Transfected explants were placed adaxial side up onto MS0 cocultivation media, and cultured at 24°C in the dark for 48 h. The plant materials were transferred to MS1 selection media, and cultured under a fluorescent light/dark cycle of 16/8 hours at 24°C for four weeks. The callus tissues were transferred to MS2 differentiation media, and cultured under a fluorescent light/dark cycle of 16/8 hours at 24°C for five weeks till the regenerated shoots were visible. Seedlings were cut off and placed on MS3 media for root induction. After cultured for five weeks, the primary and lateral roots were developed, and the seedlings were transferred to soil made up of a 3:1:1 combination of peat moss, perlite, and vermiculite (v/v/v). After cultured for two weeks under a fluorescent light cycle of 16/8 hours and a temperature of 26°C, leaves of T_0_ plants transformed with the pGK-IBC or empty vectors were sampled and subjected to further analysis. The concentration of antibiotics and phytohormones in plant culture media was listed in **Supplementary Table 2**.

### Reverse transcription-PCR

*GmCHS8*, *GmCHR5*, and *SfFPT* genes were amplified by using KOD plus DNA polymerase (Toyobo, Tokyo, Japan). Diluted cDNA from tobacco leaves agroinfiltrated with pGK-IBC was used as templates, and that with the empty vector was used as negative control. Primers for cloning were used for reverse transcription-PCR (RT-PCR). *ACTIN1* gene from tobacco (*NtACTIN1*, AB158612) was used as an endogenous reference. PCR amplification was carried out using the following thermal cycle conditions: initial denaturation at 94°C for 2min; 30 cycles of denaturation at 98°C for 10s, annealing at 60°C for 30s, and extension at 68°C for 1min. Amplicons were analyzed on a 1.2% agarose gel with 1×TAE and run at 100 V, the gel was stained with GelRed (Biotium, CA, USA).

### Quantitative real-time reverse transcription-PCR

Three leaves were collected from each pGK-IBC transgenic T_0_ line (line #1, #2, #4, #5, and #6) and an empty vector transgenic T_0_ line (line #1). Total RNA was extracted, and first-stand cDNA was synthesized from 450 ng total RNA as described above. qRT-PCR was performed by using a PerfectStart Green qPCR Master Mix (TransGen Biotech, Beijing, China) on a QuantStudio 3 Real-Time PCR System (Applied Biosystems, Foster City, CA, USA). The relative expression levels of *GmCHS8*, *GmCHR5*, and *SfFPT* were detected by the method of ΔΔCt using *NtACTIN1* as the reference gene, and presented as the mean ± standard error of three biological replicates with three technical replicates. The primers used was listed in **Supplementary Table 1**.

### Flavonoids extraction and analysis

Freeze-dried tobacco leaves were ground into powder. Approximately 500 mg dry powder was extracted with 80% methanol (v/v) by ultrasonic for 30min. After methanol was evaporated under nitrogen gas, the residue was re-dissolved in 2 ml 80% methanol and analyzed using ultra-performance liquid chromatography-quadrupole time-of-flight mass spectrometry (UPLC-Q-TOF). For each analysis, five biological replicates were applied.

A 3 μl injection of each sample was made onto ACQUITY UPLC C18 1.7 μm (2.1 × 50 mm) Column (Waters, USA) and analyzed by liquid chromatography-electrospray ionization tandem mass spectrometry (LC/ESI/MS) using an Agilent 1290 UPLC at 254 nm coupled with an Agilent Q-TOF 6560 mass spectrometer system. Data acquisition was performed in positive ion mode with a scan range of 20-750 Da. ESI source parameters were set as following: capillary voltage, 3500 V; drying gas temperature, 225°C; drying gas flow rate, 5 L/min; sheath gas temperature, 400°C; sheath gas flow rate, 12 L/min; nebulizer pressure, 20 psi; nozzle voltage, 500 V; data acquisition frequency, 1 spectrum/s. MS/MS spectra were used to obtain fragments and identify compounds. The solvents were acetonitrile (solvent A) and water containing 0.1% formic acid (solvent B) at the flow rate of 0.3 ml/min. The gradient of the solvent was: 0–5min, 3%–10% solvent A; 5–20 min, 10%–70% solvent A; 20–27 min, 70%–90% solvent A; 27–30min, 97% solvent A.

## Results

### *In planta* screening of prenyltransferase for the biosynthesis of isobavachalcone

Four prenyltransferases (GuILDT, HlPT1, SfILDT, and SfFPT) were subjected to a screening to verify whether they could convert isoliquiritigenin to IBC, by using the tobacco transient expression system. Single expression vectors of each gene were transformed into tobacco leaves, and aqueous solution of isoliquiritigenin was supplied to serve as an exogenous substrate. IBC was then detected by UPLC-Q-TOF.

**Figure 2:**
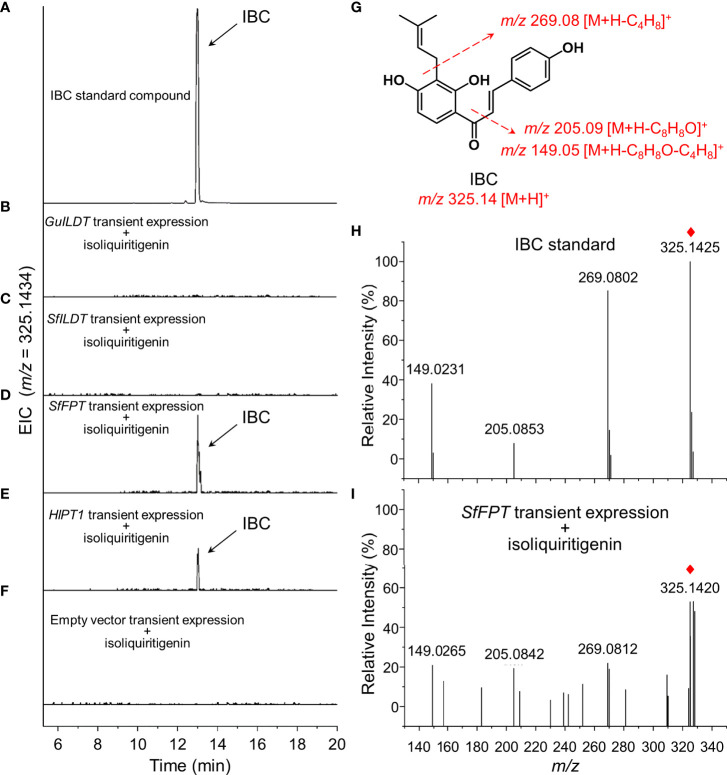
Screening of prenyltransferases for the conversion of isoliquiritigenin to isobavachalcone by transient expression. Four prenyltransferases were transiently expressed in tobacco leaves supplied with isoliquiritigenin as the substrate, to validate their *in planta* activities for the biosynthesis of isobavachalcone (IBC). Representative extracted ion chromatogram (EIC) of **(A)** IBC standard, **(B)** *GuILDT*, **(C)** *SfILDT*, **(D)** *SfFPT*, **(E)** *HlPT1* expressed samples, and **(F)** negative control was shown. **(G)** The structure of IBC and its fragmentation pattern was proposed by dotted arrows. Positive ion modes of MS/MS spectra of **(H)** IBC standard and **(I)** *SfFPT* expressed tobacco leaves was shown. Red rhombs indicated the precursor ions of IBC.

The sample of IBC standard compound showed a peak with the retention time of 13.0 min (**Figure 2A**). Although the previous *in vitro* assay demonstrated that GuILDT catalyzed the formation of IBC from isoliquiritigenin (Li et al., 2018), our *in planta* experiment showed that GuILDT failed to produce IBC by transient expression in tobacco leaves when isoliquiritigenin was supplied as the substrate (**Figure 2B**; **Table 1**). The same result was observed when *SfILDT* was agroinfiltrated (**Figure 2C**; **Table 1**). By contrast, when *SfFPT* or *HlPT1* was applied to transient expression, a new peak with the same retention time as IBC standard was observed (**Figures 2D, E**; **Table 1**). This peak was verified by UPLC-Q-TOF analysis in the electrospray ionization (ESI)-positive ion mode. The main fragments of a molecular ion peak with a mass-to-charge ratio (*m/z*) of 325.1420 [M+H]^+^ are *m/z* 269.0812 [M+H]^+^, *m/z* 205.0842 [M+H]^+^, and *m/z* 149.0265 [M+H]^+^, which was consistence with the IBC standard (**Figures 2G–I**). By contrast, when an empty vector was transiently expressed, or a prenyltransferase gene was expressed without isoliquiritigenin supplement, this peak was not detected (**Figure 2F**; **Table 1**). These results suggested that SfFPT and HlPT1 were able to convert exogenous isoliquiritigenin to IBC in the transient expression system. Quantitative analysis revealed that the concentration of IBC in *SfFPT*-infiltrated samples was significantly higher than that in *HlPT1*-infiltrated samples, indicating stronger catalytic activity of SfFPT (**Table 1**).

**Table 1:** *In planta* conversion of isoliquiritigenin to isobavachalcone by prenyltransferases using tobacco transient expression approaches.

Transgene	Substrate	Concentration of IBC
*GuILDT*	isoliquiritigenin	N.D.
	none	N.D.
*SfILDT*	isoliquiritigenin	N.D.
	none	N.D.
*SfFPT*	isoliquiritigenin	0.54 ± 0.08
	none	N.D.
*HlPT1*	isoliquiritigenin	0.27 ± 0.09
	none	N.D.
Empty vector	isoliquiritigenin	N.D.
	none	N.D.

Values shown are the concentration of isobavachalcone (IBC) (μg/g dry weight). Values are the mean of five biological replicates ± standard error. N.D., not detected.

### Re-construction of isoliquiritigenin biosynthesis pathway in tobacco

**Figure 3:**
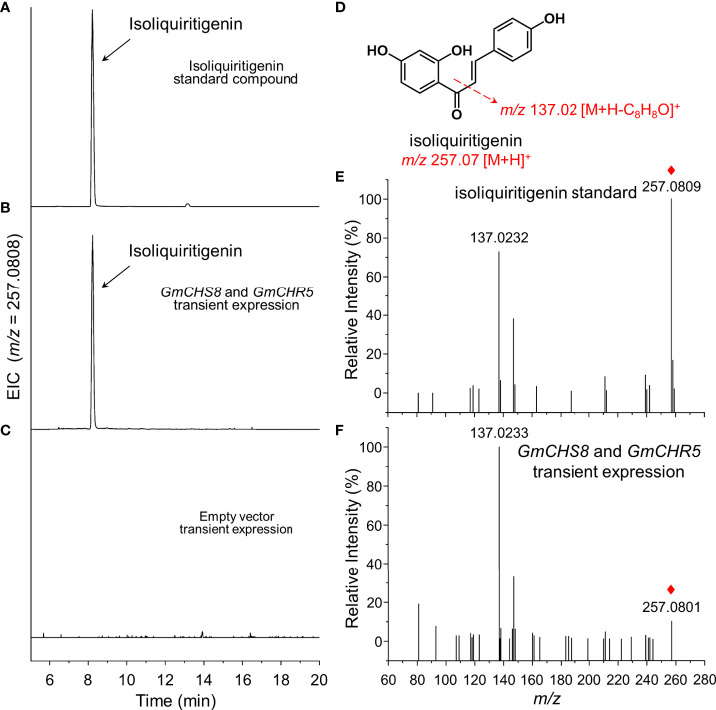
Generation of isoliquiritigenin in tobacco by transient expression. *GmCHS8* and *GmCHR5* was transiently co-expressed in tobacco leaves. Representative extracted ion chromatogram (EIC) of **(A)** isoliquiritigenin standard, **(B)** *GmCHS8* and *GmCHR5* co-expressed samples, and **(C)** negative control was shown. **(D)** The structure of isoliquiritigenin and its fragmentation pattern was proposed by dotted arrows. Positive ion modes of MS/MS spectra of **(E)** isoliquiritigenin standard and **(F)** *GmCHS8* and *GmCHR5* co-expressed samples was shown. Red rhombs indicated the precursor ions of isoliquiritigenin.

Two single gene expression vectors carrying *GmCHS8* and *GmCHR5* were constructed and co-expressed by transient expression in tobacco leaves as mentioned above. The samples were subjected to UPLC-Q-TOF analysis. The presence of chromatographic peaks with an identical retention time as the isoliquiritigenin standard was observed (**Figures 3A, B**). When an empty vector was transiently expressed, this peak was not detected (**Figure 3C**). QTOF-MS/MS analysis revealed that the sample showed MS ion of *m/z* 257.0801 [M+H]^+^ and fragments of *m/z* 137.0233 [M+H]^+^, which was identical as the isoliquiritigenin standard (**Figures 3D–F**). This result indicated that the heterologous biosynthesis of isoliquiritigenin in tobacco was achieved by introducing *GmCHS8* and *GmCHR5*. Quantitative analysis showed that the samples generated isoliquiritigenin with concentrations around 21.0 μg/g.

### Construction of multigene expression vectors

**Figure 4:**
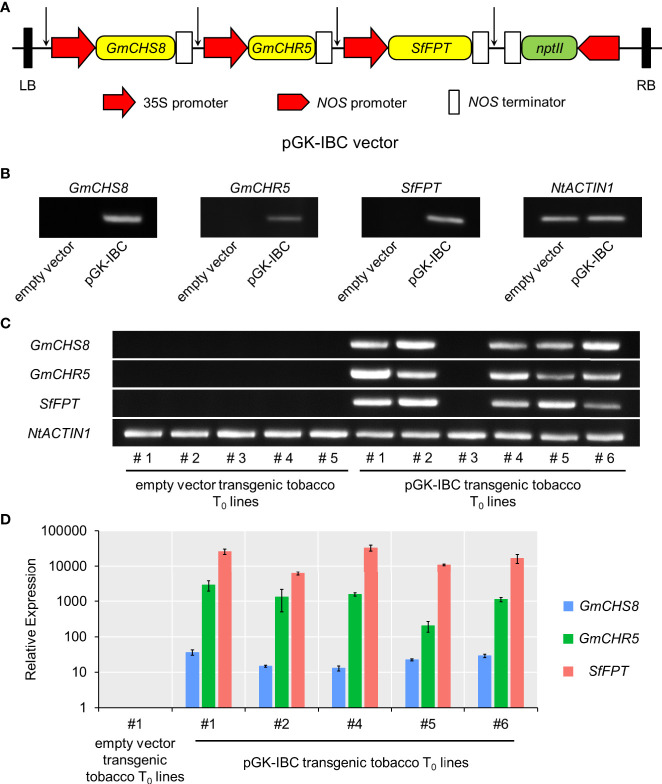
Schematic structure of the multigene expression vector and the validation of its gene expression. **(A)** The region spanning the LB and RB was indicated. Arrows indicated the conjunction sites after Golden Gate assembly. **(B)** RT-PCR analysis of *GmCHS8*, *GmCHR5*, and *SfFPT* in pKG-IBC and empty vectors transiently expressed tobacco leaves. **(C)** RT-PCR analysis of pGK-IBC and empty vectors transgenic tobacco T_0_ lines. The expression of *GmCHS8*, *GmCHR5*, and *SfFPT* was confirmed. *NtACTIN1* was used as a reference gene. **(D)** Quantitative real-time reverse transcription-PCR analysis of pGK-IBC and empty vectors transgenic tobacco T_0_ lines. LB, left border; RB, right border; *nptII*, *neomycin phosphotransferase*.

To establish the genetic transformation of tobacco producing IBC, a multigene expression vector carrying three selected genes, i.e., *GmCHS8*, *GmCHR5*, and *SfFPT*, was constructed. The promoter-CDS-terminator cassettes of each gene were amplified and assembled into an pPZP vector backbone (Hajdukiewicz et al., 1994) based on Golden Gate assembly protocol (Engler et al., 2008). In the resulting multigene expression vector (named as pGK-IBC), three genes were tandemly arranged, and their expression was independently control by their own promoter and terminator (**Figure 4A**). The pGK-IBC and empty vectors were transformed into *Agrobacterium* GV3101. Tobacco leaves were transformed with the pGK-IBC or empty vectors by transient expression. RT-PCR analysis showed the bands in samples transformed with the pGK-IBC vector, but no in those transformed with the empty vector, indicating that all genes in the pGK-IBC vector could be transcribed in planta (**Figure 4B**).

### *De novo* biosynthesis of IBC in transgenic tobacco

Subsequently, the pGK-IBC and empty vectors were genetically introduced into wild-type tobacco plants *via Agrobacterium* infection. Six and five seedlings transformed with the pGK-IBC and empty vectors exhibited kanamycin resistance, respectively (**Supplementary Figure 2**). Five independent transgenic (T_0_) plants expressing *GmCHS8*, *GmCHR5*, and *SfFPT*, genes were confirmed by RT-PCR (**Figure 4C**). Quantitative real-time reverse transcription-PCR analysis showed that the expression levels of these genes were dramatically increased in five independent pGK-IBC transgenic T_0_ plants when compared with those in an empty vector transgenic T_0_ line (**Figure 4D**). No obvious morphological difference was observed between the pGK-IBC and empty vector transformants (**Supplementary Figure 3**).

Five pGK-IBC and three empty vector transgenic tobacco T_0_ lines were subjected to the UPLC-Q-TOF analysis. The appearance of IBC was detected by peaks with the identical retention time of IBC standard (**Supplementary Figures 4A–C**), and confirmed by mass spectra with the MS ion of *m/z* 325.1432 [M+H]^+^, fragments of *m/z* 269.0810 [M+H]^+^, *m/z* 205.0878 [M+H]^+^, and *m/z* 149.0233 [M+H]^+^ (**Supplementary Figure 5**). The pGK-IBC transgenic line #1 produced the highest amount of IBC up to 0.56 μg/g (DW) (**Table 2**). The presence of isoliquiritigenin in pGK-IBC transgenic seedlings was also detected with the concentration ranging from 11.0 to 12.3 μg/g (DW) (**Table 2**; **Supplementary Figures 4D–F**). It is noticeable that the contents of IBC generated in transgenic plants was relative to that in the *SfFPT* transient expression experiment, in which sufficient isoliquiritigenin was supplied as the substrate, indicating that low yield of IBC was not attributed to the substrate supplement.

**Table 2:** The accumulation of IBC and isoliquiritigenin in seedlings of transgenic tobacco T_0_ lines.

Transformants	Lines	IBC	Isoliquiritigenin
pGK-IBC transgenic tobacco	#1	0.56 ± 0.06	11.5 ± 0.9
	#2	0.52 ± 0.11	12.3 ± 1.3
	#4	0.46 ± 0.05	12.1 ± 0.8
	#5	0.49 ± 0.03	11.7 ± 2.5
	#6	0.55 ± 0.12	11.0 ± 1.4
Empty vector transgenic tobacco	#1	N.D.	N.D.
	#2	N.D.	N.D.
	#3	N.D.	N.D.

Values shown are the concentration of isobavachalcone (IBC) and liquiritigenin (μg/g dry weight). Values are the mean of five biological replicates ± standard error. N.D., not detected.

## Discussion

In this study, we perform an *in planta* screening of previously reported prenyltransferases for the biosynthesis of IBC, and construct the *de novo* heterologous biosynthesis of IBC in tobacco by introducing *GmCHS8*, *GmCHR5*, and *SfFPT*. An interesting issue raised from our results is the different catalytic properties of these prenyltransferases from previous studies. At present, about 17 flavonoid pathway-specific prenyltransferases have been reported, and the *in vitro* activities of most of them have been well characterized by yeast recombinant proteins. By contrast, their *in vivo* activities were poorly mentioned, including four prenyltransferases we selected in this study. When *GuILDT* was expressed in yeast, their microsome extraction containing the recombinant GuILDT enzymes was able to convert isoliquiritigenin to IBC (Li et al., 2018). However, when *GuILDT* was expressed with isoliquiritigenin in tobacco in our study, no IBC accumulation was observed (**Figure 2B**; **Table 1**). Such *in vivo* and *in vitro* difference seems to be not occasional in prenyltransferase studies, since the functions of SfN8DT (Sasaki et al., 2008) and LaPT2 (Liu et al., 2021) were reported to be different between transgenic *Arabidopsis thaliana* and recombinant proteins. This phenomenon is probably caused by different microenvironments *in vitro* and *in planta*, including substrate concentration and accessibility, subcellular localization of enzymes, co-factors, competitive inhibition of enzyme activity, etc. Therefore, when a prenyltransferase is applied for plant-based metabolic engineering, its *in planta* function should be carefully confirmed. Fortunately, two prenyltransferases showed the activity to convert isoliquiritigenin to IBC in tobacco in our screening (**Figures 2D, E**; **Table 1**). The substrate specificity of SfFPT (Chen et al., 2013) and HlPT1 (Tsurumaru et al., 2012) towards liquiritigenin and naringenin chalcone, respectively, was demonstrated by using recombinant proteins, whereas isoliquiritigenin was not checked in these studies. Based on the structural similarity of isoliquiritigenin to liquiritigenin and naringenin chalcone, it would be reasonable that SfFPT and HlPT1 can catalyze the prenylation of isoliquiritigenin in tobacco.

Although the *de novo* biosynthesis of IBC was achieved in our transgenic tobacco plants, its concentration was not satisfying. Generally, low production rate in metabolic engineering is attributed to the insufficient substrate supplement. However, sufficient supplement of isoliquiritigenin in our transient expression experiment (**Table 1**) could not increase the accumulation of IBC when compared with the *de novo* biosynthesis of IBC (**Table 2**), suggesting that the poor yield of IBC was caused by the low activity of SfFPT enzyme. SfFPT is isolated from *S. flavescens*, in which IBC is not the predominant prenylflavonoids and the level of IBC is extremely low. Therefore, the major activity of SfFPT in *S. flavescens* is probably not subjected to isoliquiritigenin. In this respect, further identification of isoliquiritigenin-specific prenyltransferases in some IBC-producing plants, such as *P. corylifolia* and *Erythrina variegata*, is expected to improve the IBC production rate in metabolic engineering.

Another fact is that prenyltransferases have putative transmembrane domains and are localized in plastids (Chang et al., 2021). In previous attempts to produce prenylflavonoid in transgenic plants, overexpression of prenyltransferases in the plastid exhibited the highest activities than that in the cytosol and mitochondria (Koeduka et al., 2011; Sugiyama et al., 2011). However, the flavonoid biosynthetic enzyme complexes (Nakayama et al., 2019), as well as the flavonoids as substrates, are localized in the endoplasmic reticulum. The subcellular compartment of prenylflavonoid enzymes inhibits the substrate accessibility of parent flavonoids with prenyltransferases, and thus limits the efficiency of prenylation of flavonoids. An alternative strategy is to re-construct the IBC biosynthesis pathway in the chloroplast. The crowded microenvironment in the chloroplast consisting of membrane and stroma may shorten the distance between prenyltransferases and other flavonoid biosynthetic enzymes, thereby enhance the efficiency of IBC biosynthesis. Abundant phenylalanine is also provided as a substrate of the flavonoid pathway without transport to the cytosol. More important, lots of enzymes involved in the biosynthesis and secondary modification of flavonoids exist in the cytosol, which may consume the target products *via* competitive or non-specific reactions. As an organelle for primary and energy metabolism, the chloroplast possesses relatively “pure” environment for secondary metabolism. Re-construction of flavonoid pathway in the chloroplast can avoid undesirable reaction. Recently, the biosynthesis pathway of dhurrin and precursors of taxane has been constructed in the tobacco chloroplast (Gnanasekaran et al., 2016; Li et al., 2019b). It will be interesting to construct the biosynthesis pathway of prenylflavonoid in the chloroplast.

Although IBC exhibits excellent pharmacological effects such as antibacterial and antiviral activities, it has not been commercially used as yet due to the difficult availability based on natural plant extraction. The current price of IBC is around 25,000 USD/kg in the market of China (data were collected by telephone enquiry from four companies in China). Our study provided a strategy to produce high valuable natural compounds. However, several critical challenges are raised. The metabolic background in the plant cells is more complicated than that in the microorganisms. As shown in this study, genes with identified functions *via in vitro* experiments might play different roles in plant cells. Numerous enzymes in the plant cytosol may also direct the metabolic flux to branch pathways, or catalyze the target compound to undesired derivatives. In addition, the gene manipulation in plants is more difficult than that in microorganism. For stable genetic transformation of plants, selected genes have to be transformed simultaneously by constructing multigene expression vectors. Despite all these challenges, once an ideal yield of the target compound is achieved in the plant-based metabolic engineering, a much lower cost of production is excepted due to its electricity-free and non-pollution properties, even compared with the microorganism-based fermentation.

## Data availability statement

The original contributions presented in the study are included in the article/**Supplementary Material**. Further inquiries can be directed to the corresponding authors.

## Author contributions

LG, HC, and YL conceived and designed the experiments; LG, WZ, YW, and YY performed the experiments; LG, CW, and JG analyzed the data; LG, ZY, and YL prepared the manuscript; JD, AB, and MYH provided advice on the experiments and revised the manuscript; YL, HC, and MYH provided the fundings. All authors read and approved the final manuscript.

## Fundings

This work was supported by the Fundamental Research Funds for the Central Universities (lzujbky-2020-46) to YL, the Qinghai Province International Cooperation Project (2021-HZ-805) to YL, HC, and MYH, Qinghai Province Applied Basic Project (2020-ZJ-743) to HC, and Kunlun Talent·High-end Innovation and Entrepreneurship Talents in Qinghai Provience (2020).

## Acknowledgments

We thank Prof. Yingqian Liu from School of Pharmacy, Lanzhou University for providing the standard compounds, and Dr. Na Yan from School of Pharmacy, Lanzhou University for providing technical assistance using LC-Q-TOF in Medical Experimental Center of Lanzhou University.

## Conflict of interest

The authors declare that the research was conducted in the absence of any commercial or financial relationships that could be construed as a potential conflict of interest.

## Publisher’s note

All claims expressed in this article are solely those of the authors and do not necessarily represent those of their affiliated organizations, or those of the publisher, the editors and the reviewers. Any product that may be evaluated in this article, or claim that may be made by its manufacturer, is not guaranteed or endorsed by the publisher.

## Abbreviations

IBC, isobavachalcone; RT-PCR, reverse transcription polymerase chain reaction; EIC, extracted ion chromatogram; UPLC-Q-TOF, ultra-performance liquid chromatography-quadrupole time-of-flight mass spectrometry.

## References

[B1] Ahrazem O. Zhu C. Huang X. Rubio-Moraga A. Capell T. Christou P.. (2022). Metabolic engineering of crocin biosynthesis in *Nicotiana species* . Front. Plant Sci. 13. doi: 10.3389/fpls.2022.861140 PMC8957871 35350302

[B2] Ahuja I. Kissen R. Bones A. M. (2012). Phytoalexins in defense against pathogens. Trends Plant Sci. 17, 73–90. doi: 10.1016/j.tplants.2011.11.002 22209038

[B3] Akram M. Rasool A. An T. Feng X. Li C. (2021). Metabolic engineering of *Yarrowia lipolytica* for liquiritigenin production. Chem. Eng. Sci. 230, 116177. doi: 10.1016/j.ces.2020.116177

[B4] Allen Q. M. Febres V. J. Rathinasabapathi B. Chaparro J. X. (2022). Engineering a plant-derived astaxanthin synthetic pathway into *Nicotiana benthamiana* . Front. Plant Sci. 12. doi: 10.3389/fpls.2021.831785 PMC8804313 35116052

[B5] An B. H. Jeong H. Zhou W. Liu X. Kim S. Jang C. Y.. (2016). Evaluation of the biological activity of opuntia ficus indica as a tissue- and estrogen receptor subtype-selective modulator. Phytother. Res. 30, 971–980. doi: 10.1002/ptr.5602 26989859

[B6] Bell N. Molloy J. E. (2022). Efficient golden gate assembly of DNA constructs for single molecule force spectroscopy and imaging. Nucleic Acids Res. 50, e77. doi: 10.1093/nar/gkac300 35489063 PMC9303394

[B7] Botta B. Delle Monache G. Menendez P. Boffi A. (2005a). Novel prenyltransferase enzymes as a tool for flavonoid prenylation. Trends Pharmacol. Sci. 26, 606–608. doi: 10.1016/j.tips.2005.09.012 16229901

[B8] Botta B. Vitali A. Menendez P. Misiti D. Delle Monache G. (2005b). Prenylated flavonoids: pharmacology and biotechnology. Curr. Med. Chem. 12, 717–739. doi: 10.2174/0929867053202241 15790308

[B9] Chang H. Y. Cheng T. H. Wang A. H. (2021). Structure, catalysis, and inhibition mechanism of prenyltransferase. IUBMB Life 73, 40–63. doi: 10.1002/iub.2418 33246356 PMC7839719

[B10] Chen Q. Li Y. Chen Z. (2012). Separation, identification, and quantification of active constituents in fructus psoraleae by high-performance liquid chromatography with UV, ion trap mass spectrometry, and electrochemical detection. J. Pharm. Anal. 2, 143–151. doi: 10.1016/j.jpha.2011.11.005 29403734 PMC5760910

[B11] Chen R. Liu X. Zou J. Yin Y. Ou B. Li J.. (2013). Regio- and stereospecific prenylation of flavonoids bySophora flavescensPrenyltransferase. Adv. Synth. Catal. 355, 1817–1828. doi: 10.1002/adsc.201300196

[B12] Dzoyem J. P. Nkuete A. H. L. Ngameni B. Eloff J. N. (2017). Anti-inflammatory and anticholinesterase activity of six flavonoids isolated from polygonum and dorstenia species. Arch. Pharm. Res. 40, 1129–1134. doi: 10.1007/s12272-015-0612-9 26048035

[B13] Engler C. Kandzia R. Marillonnet S. (2008). A one pot, one step, precision cloning method with high throughput capability. Plos One 3, e3647. doi: 10.1371/journal.pone.0003647 18985154 PMC2574415

[B14] Gao Q. Xu Z. Zhao G. Wang H. Weng Z. Pei K.. (2016). Simultaneous quantification of 5 main components of psoralea corylifolia l. @ in rats' plasma by utilizing ultra high pressure liquid chromatography tandem mass spectrometry. J. Chromatogr. B 1011, 128–135. doi: 10.1016/j.jchromb.2015.12.044 26773881

[B15] Gester S. Metz P. Zierau O. Vollmer G. (2001). An efficient synthesis of the potent phytoestrogens 8-prenylnaringenin and 6-(1,1-dimethylallyl)naringenin by europium(III)-catalyzed claisen rearrangement. Tetrahedron 57, 1015–1018. doi: 10.1016/s0040-4020(00)01078-4

[B16] Gnanasekaran T. Karcher D. Nielsen A. Z. Martens H. J. Ruf S. Kroop X.. (2016). Transfer of the cytochrome P450-dependent dhurrin pathway from sorghum bicolor into nicotiana tabacum chloroplasts for light-driven synthesis. J. Exp. Bot. 67, 2495–2506. doi: 10.1093/jxb/erw067 26969746 PMC4809297

[B17] Gomes D. Rodrigues L. R. Rodrigues J. L. (2022). Perspectives on the design of microbial cell factories to produce prenylflavonoids. Int. J. Food Microbiol. 367, 109588. doi: 10.1016/j.ijfoodmicro.2022.109588 35245724

[B18] Grealis J. P. Müller-Bunz H. Ortin Y. Casey M. Mcglinchey M. J. (2013). Synthesis of isobavachalcone and some organometallic derivatives. Eur. J. Org. Chem. 2013, 332–347. doi: 10.1002/ejoc.201201063

[B19] Grienke U. Richter M. Walther E. Hoffmann A. Kirchmair J. Makarov V.. (2016). Discovery of prenylated flavonoids with dual activity against influenza virus and streptococcus pneumoniae. Sci. Rep. 6, 27156. doi: 10.1038/srep27156 27257160 PMC4891693

[B20] Hajdukiewicz P. Svab Z. Maliga P. (1994). The small, versatile pPZP family of agrobacterium binary vectors for plant transformation. Plant Mol. Biol. 25, 989–994. doi: 10.1007/bf00014672 7919218

[B21] Hasunuma T. Miyazawa S. Yoshimura S. Shinzaki Y. Tomizawa K. Shindo K.. (2008). Biosynthesis of astaxanthin in tobacco leaves by transplastomic engineering. Plant J. 55, 857–868. doi: 10.1111/j.1365-313X.2008.03559.x 18494855

[B22] Horsch R. B. Fry J. E. Hoffmann N. L. Eichholtz D. Rogers S. G. Fraley R. T. (1985). A simple and general method for transferring genes into plants. Science 227, 1229–1231. doi: 10.1126/science.227.4691.1229 17757866

[B23] Koeduka T. Shitan N. Kumano T. Sasaki K. Sugiyama A. Linley P.. (2011). Production of prenylated flavonoids in tomato fruits expressing a prenyltransferase gene from *Streptomyces coelicolor* A3(2). Plant Biol. 13, 411–415. doi: 10.1111/j.1438-8677.2010.00409.x 21309988

[B24] Kretzschmar G. Zierau O. Wober J. Tischer S. Metz P. Vollmer G. (2010). Prenylation has a compound specific effect on the estrogenicity of naringenin and genistein. J. Steroid Biochem. Mol. Biol. 118, 1–6. doi: 10.1016/j.jsbmb.2009.08.005 19733663

[B25] Lau W. Sattely E. S. (2015). Six enzymes from mayapple that complete the biosynthetic pathway to the etoposide aglycone. Science 349, 1224–1228. doi: 10.1126/science.aac7202 26359402 PMC6861171

[B26] Levisson M. Araya-Cloutier C. De Bruijn W. J. C. van der Heide M. Salvador Lopez J. M. Daran J. M.. (2019). Toward developing a yeast cell factory for the production of prenylated flavonoids. J. Agric. Food Chem. 67, 13478–13486. doi: 10.1021/acs.jafc.9b01367 31016981 PMC6909231

[B27] Li J. Chen R. Wang R. Liu X. Xie K. Chen D.. (2018). Biocatalytic access to diverse prenylflavonoids by combining a regiospecific c-prenyltransferase and a stereospecific chalcone isomerase. Acta Pharm. Sin. B 8, 678–686. doi: 10.1016/j.apsb.2018.01.009 30109191 PMC6089845

[B28] Li J. Mutanda I. Wang K. Yang L. Wang J. Wang Y. (2019b). Chloroplastic metabolic engineering coupled with isoprenoid pool enhancement for committed taxanes biosynthesis in nicotiana benthamiana. Nat. Commun. 10, 4850. doi: 10.1038/s41467-019-12879-y 31649252 PMC6813417

[B29] Liu R. Hu Y. Li J. Lin Z. (2007). Production of soybean isoflavone genistein in non-legume plants *via* genetically modified secondary metabolism pathway. Metab. Eng. 9, 1–7. doi: 10.1016/j.ymben.2006.08.003 17029902

[B30] Liu J. Xia Y. Jiang W. Shen G. Pang Y. (2021). LaPT2 gene encodes a flavonoid prenyltransferase in white lupin. Front. Plant Sci. 12. doi: 10.3389/fpls.2021.673337 PMC8226212 34177989

[B31] Li B. Xu N. Wan Z. Ma L. Li H. Cai W.. (2019a). Isobavachalcone exerts antiproliferative and proapoptotic effects on human liver cancer cells by targeting the ERKs/RSK2 signaling pathway. Oncol. Rep. 41, 3355–3366. doi: 10.3892/or.2019.7090 30942462

[B32] Lu Y. Stegemann S. Agrawal S. Karcher D. Ruf S. Bock R. (2017). Horizontal transfer of a synthetic metabolic pathway between plant species. Curr. Biol. 27, 3034–3041.e3. doi: 10.1016/j.cub.2017.08.044 28943084

[B33] Mameda R. Waki T. Kawai Y. Takahashi S. Nakayama T. (2018). Involvement of chalcone reductase in the soybean isoflavone metabolon: identification of GmCHR5, which interacts with 2-hydroxyisoflavanone synthase. Plant J. 96, 56–74. doi: 10.1111/tpj.14014 29979476

[B34] Mann V. Harker M. Pecker I. Hirschberg J. (2000). Metabolic engineering of astaxanthin production in tobacco flowers. Nat. Biotechnol. 18, 888–892. doi: 10.1038/78515 10932161

[B35] Martí M. Diretto G. Aragonés V. Frusciante S. Ahrazem O. Gómez-Gómez L.. (2020). Efficient production of saffron crocins and picrocrocin in *Nicotiana benthamiana* using a virus-driven system. Metab. Eng. 61, 238–250. doi: 10.1016/j.ymben.2020.06.009 32629020

[B36] Mortimer C. L. Misawa N. Perez-Fons L. Robertson F. P. Harada H. Bramley P. M.. (2017). The formation and sequestration of nonendogenous ketocarotenoids in transgenic *Nicotiana glauca* . Plant Physiol. 173, 1617–1635. doi: 10.1104/pp.16.01297 28153925 PMC5338661

[B37] Mukai R. (2018). Prenylation enhances the biological activity of dietary flavonoids by altering their bioavailability. Biosci. Biotechol. Biochem. 82, 207–215. doi: 10.1080/09168451.2017.1415750 29307271

[B38] Nakagawa T. Kurose T. Hino T. Tanaka K. Kawamukai M. Niwa Y.. (2007). Development of series of gateway binary vectors, pGWBs, for realizing efficient construction of fusion genes for plant transformation. J. Biosci. Bioeng. 104, 34–41. doi: 10.1263/jbb.104.34 17697981

[B39] Nakayama T. Takahashi S. Waki T. (2019). Formation of flavonoid metabolons: functional significance of protein-protein interactions and impact on flavonoid chemodiversity. Front. Plant Sci. 10. doi: 10.3389/fpls.2019.00821 PMC6629762 31338097

[B40] Nemoto H. Kawamura T. Hayashi M. Mukai R. Terao J. (2012). An efficient method for C8-prenylation of flavonols and flavanones. Synthesis 44, 1308–1314. doi: 10.1055/s-0031-1290756

[B41] Nett R. S. Lau W. Sattely E. S. (2020). Discovery and engineering of colchicine alkaloid biosynthesis. Nature 584, 148–153. doi: 10.1038/s41586-020-2546-8 32699417 PMC7958869

[B42] Pandey A. Misra P. Khan M. P. Swarnkar G. Tewari M. C. Bhambhani S.. (2014). Co-Expression of *Arabidopsis* transcription factor, AtMYB12, and soybean isoflavone synthase, GmIFS1, genes in tobacco leads to enhanced biosynthesis of isoflavones and flavonols resulting in osteoprotective activity. Plant Biotechnol. J. 12, 69–80. doi: 10.1111/pbi.12118 24102754

[B43] Ralston L. Subramanian S. Matsuno M. Yu O. (2005). Partial reconstruction of flavonoid and isoflavonoid biosynthesis in yeast using soybean type I and type II chalcone isomerases. Plant Physiol. 137, 1375–1388. doi: 10.1104/pp.104.054502 15778463 PMC1088328

[B44] Rogers S. G. Horsch R. B. Fraley R. T. (1986). Gene transfer in plants: Production of transformed plants using Ti plasmid vectors. Meth. Enz. 188, 627–640. doi: 10.1016/0076-6879(86)18105-5

[B45] Saito K. Matsuo Y. Imafuji H. Okubo T. Maeda Y. Sato T.. (2018). Xanthohumol inhibits angiogenesis by suppressing nuclear factor-kappaB activation in pancreatic cancer. Cancer Sci. 109, 132–140. doi: 10.1111/cas.13441 29121426 PMC5765302

[B46] Sasaki K. Mito K. Ohara K. Yamamoto H. Yazaki K. (2008). Cloning and characterization of naringenin 8-prenyltransferase, a flavonoid-specific prenyltransferase of sophora flavescens. Plant Physiol. 146, 1075–1084. doi: 10.1104/pp.107.110544 18218974 PMC2259047

[B47] Sasaki K. Tsurumaru Y. Yamamoto H. Yazaki K. (2011). Molecular characterization of a membrane-bound prenyltransferase specific for isoflavone from sophora flavescens. J. Biol. Chem. 286, 24125–24134. doi: 10.1074/jbc.m111.244426 21576242 PMC3129193

[B48] Sastre-Serra J. Ahmiane Y. Roca P. Oliver J. Pons D. G. (2019). Xanthohumol, a hop-derived prenylflavonoid present in beer, impairs mitochondrial functionality of SW620 colon cancer cells. Int. J. Food Sci. Nutr. 70, 396–404. doi: 10.1080/09637486.2018.1540558 30458656

[B49] Schultz B. J. Kim S. Y. Lau W. Sattely E. S. (2019). Total biosynthesis for milligram-scale production of etoposide intermediates in a plant chassis. J. Am. Chem. Soc 141, 19231–19235. doi: 10.1021/jacs.9b10717 31755709 PMC7380830

[B50] Sierro N. Battey J. N. D. Ouadi S. Bakaher N. Bovet L. Willig A.. (2014). The tobacco genome sequence and its comparison with those of tomato and potato. Nat. Commun. 5, 3833–3842 doi: 10.1038/ncomms4833 24807620 PMC4024737

[B51] Song M. Liu Y. Li T. Liu X. Hao Z. Ding S.. (2021). Plant natural flavonoids against multidrug resistant pathogens. Adv. Sci. 8, e2100749. doi: 10.1002/advs.202100749 PMC8336499 34041861

[B52] Stulikova K. Karabin M. Nespor J. Dostalek P. (2018). Therapeutic perspectives of 8-prenylnaringenin, a potent phytoestrogen from hops. Molecules 23, 660. doi: 10.3390/molecules23030660 PMC6017581 29543713

[B53] Sugiyama A. Linley P. J. Sasaki K. Kumano T. Yamamoto H. Shitan N.. (2011). Metabolic engineering for the production of prenylated polyphenols in transgenic legume plants using bacterial and plant prenyltransferases. Metab. Eng. 13, 629–637. doi: 10.1016/j.ymben.2011.07.003 21835257

[B54] Tian L. Dixon R. A. (2006). Engineering isoflavone metabolism with an artificial bifunctional enzyme. Planta 224, 496–507. doi: 10.1007/s00425-006-0233-0 16482434

[B55] Tischer S. Metz P. (2007). Selective c-6 prenylation of flavonoidsvia Europium(III)- catalyzed claisen rearrangement and cross-metathesis. Adv. Synth. Catal. 349, 147–151. doi: 10.1002/adsc.200600454

[B56] Tsai W. J. Hsin W. C. Chen C. C. (1996). Antiplatelet flavonoids from seeds of psoralea corylifolia. J. Nat. Prod. 59, 671–672. doi: 10.1021/np960157y 8759164

[B57] Tsurumaru Y. Sasaki K. Miyawaki T. Uto Y. Momma T. Umemoto N.. (2012). HlPT-1, a membrane-bound prenyltransferase responsible for the biosynthesis of bitter acids in hops. Biochem. Biophys. Res. Commun. 417, 393–398. doi: 10.1016/j.bbrc.2011.11.125 22166201

[B58] Wang R. Chen R. Li J. Liu X. Xie K. Chen D.. (2014). Molecular characterization and phylogenetic analysis of two novel regio-specific flavonoid prenyltransferases from morus alba and cudrania tricuspidata. J. Biol. Chem. 289, 35815–35825. doi: 10.1074/jbc.m114.608265 25361766 PMC4276850

[B59] Wang S. Du Q. Sun J. Geng S. Zhang Y. (2022). Investigation of the mechanism of isobavachalcone in treating rheumatoid arthritis through a combination strategy of network pharmacology and experimental verification. J. Ethnopharmacol. 294, 115342. doi: 10.1016/j.jep.2022.115342 35525528

[B60] Xing N. Meng X. Wang S. (2022). Isobavachalcone: A comprehensive review of its plant sources, pharmacokinetics, toxicity, pharmacological activities and related molecular mechanisms. Phytother. Res. 36, 3120–3140. doi: 10.1002/ptr.7520 35684981

[B61] Yang X. Jiang Y. Yang J. He J. Sun J. Chen F.. (2015). Prenylated flavonoids, promising nutraceuticals with impressive biological activities. Trends Food Sci. Tech. 44, 93–104. doi: 10.1016/j.tifs.2015.03.007

[B62] Yi J. Derynck M. R. Chen L. Dhaubhadel S. (2010). Differential expression of CHS7 and CHS8 genes in soybean. Planta 231, 741–753. doi: 10.1007/s00425-009-1079-z 20016991

[B63] Yin Y. Li Y. Jiang D. Zhang X. Gao W. Liu C. (2020). *De novo* biosynthesis of liquiritin in *Saccharomyces cerevisiae* . Acta Pharm. Sin. B. 10, 711–721. doi: 10.1016/j.ces.2020.116177 32322472 PMC7161706

[B64] Yu O. Jung W. Shi J. Croes R. A. Fader G. M. McGonigle B.. (2000). Production of the isoflavones genistein and daidzein in non-legume dicot and monocot tissues. Plant Physiol. 124, 781–794. doi: 10.1104/pp.124.2.781 11027726 PMC59182

[B65] Zhao P. Inoue K. Kouno I. Yamamoto H. (2003). Characterization of leachianone G 2"-dimethylallyltransferase, a novel prenyl side-chain elongation enzyme for the formation of the lavandulyl group of sophoraflavanone G in sophora flavescens ait. cell suspension cultures. Plant Physiol. 133, 1306–1313. doi: 10.1104/pp.103.025213 14551337 PMC281625

[B66] Zhou L. Tang J. Yang X. Dong H. Xiong X. Huang J.. (2019). Five constituents in psoralea corylifolia l. attenuate palmitic acid-induced hepatocyte injury *via* inhibiting the protein kinase c-alpha/Nicotinamide-Adenine dinucleotide phosphate oxidase pathway. Front. Pharmacol. 10. doi: 10.3389/fphar.2019.01589 PMC7025552 32116659

[B67] Zhu C. Gerjets T. Sandmann G. (2007). *Nicotiana glauca* engineered for the production of ketocarotenoids in flowers and leaves by expressing the cyanobacterial crtO ketolase gene. Transgenic Res. 16, 813–821. doi: 10.1007/s11248-007-9151-6 17940844

