# Supplementary material for: Heterologous biosynthesis of isobavachalcone in tobacco based on in planta screening of prenyltransferases
Source: Front Plant Sci. 2022 Oct 6;13:1034625. doi: 10.3389/fpls.2022.1034625 (PMC9582842; doi:10.3389/fpls.2022.1034625)
Supplement: Supplementary file 1 [file DataSheet_1.docx]

Supplementary Material

# 1. Supplementary Figure and Table

## 1.1 Supplementary Figures

**
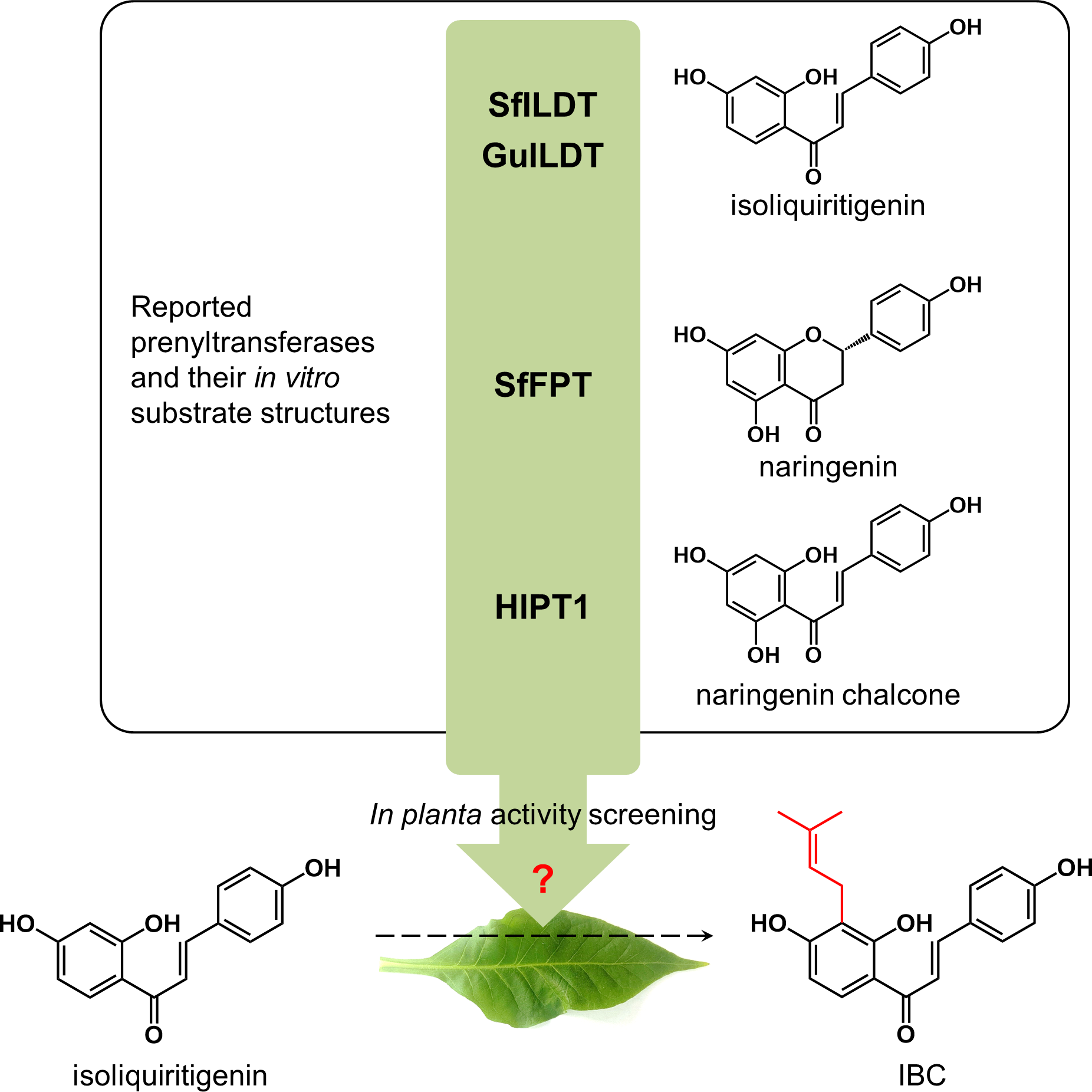
**

**Supplementary Figure 1.** The criteria for selecting prenyltransferases in this study. Four reported prenyltransferases, SfILDT, GuILDT, SfFPT, and HlPT1, were selected based on their *in vitro* substrate structures, and subjected to an *in planta* activity screening to check whether they could convert isoliquiritigenin to IBC. IBC, isobavachalcone.


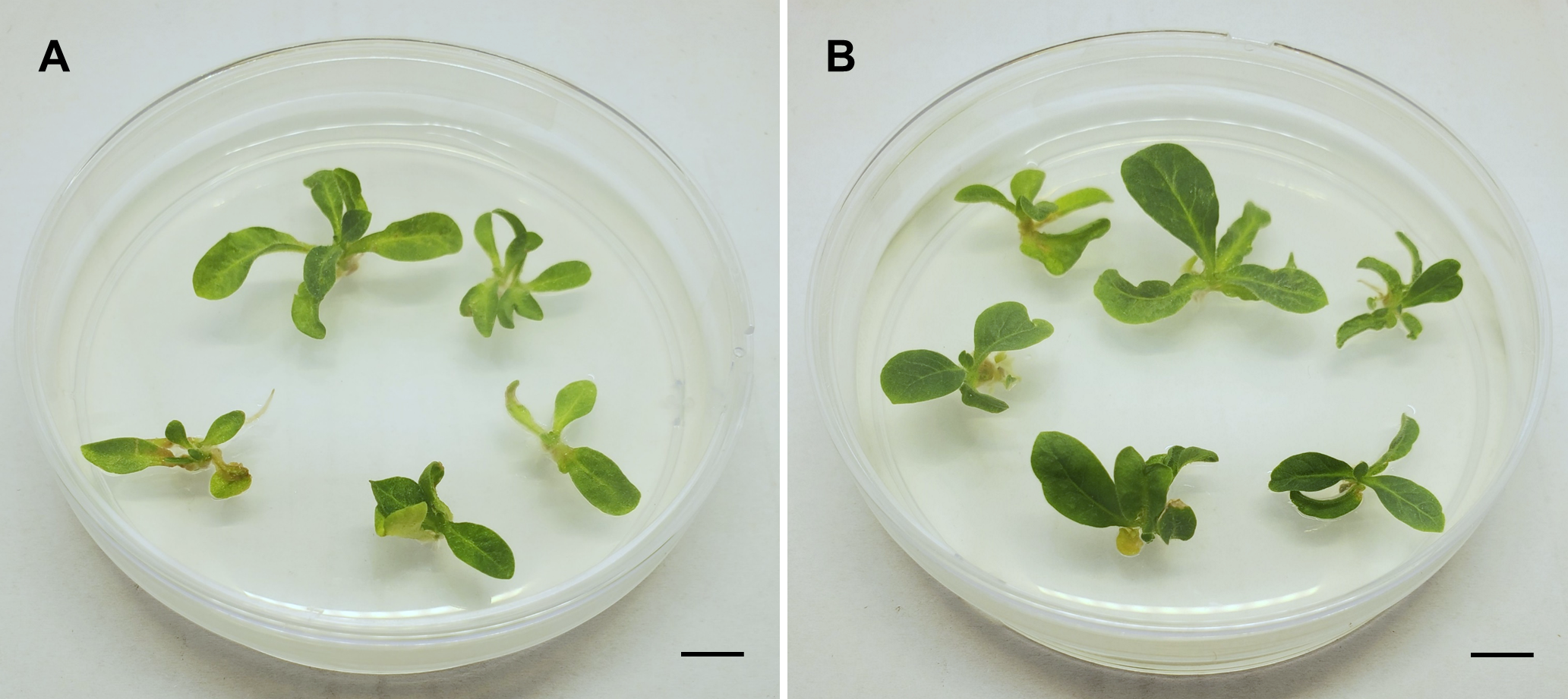


**Supplementary Figure 2.** 9-week-old seedling of (A) the empty vector and (B) pGK-IBC transgenic tobacco T_0_ lines cultured on MS3 rooting media. Scale bar = 1 cm.


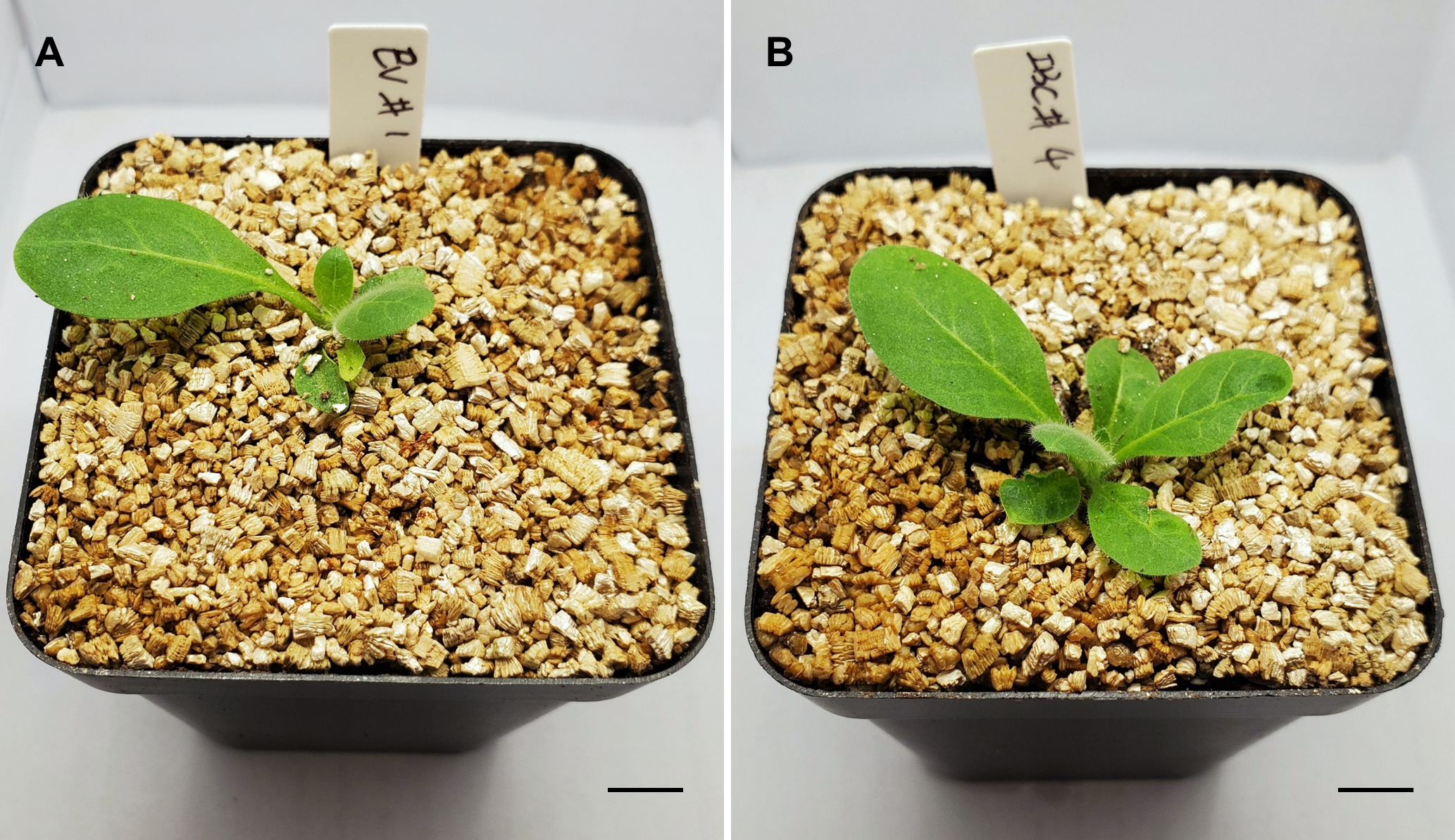


**Supplementary Figure 3.** 11-week-old seedling of (A) the empty vector and (B) pGK-IBC transgenic tobacco T_0_ lines cultured on soil. Scale bar = 1 cm.


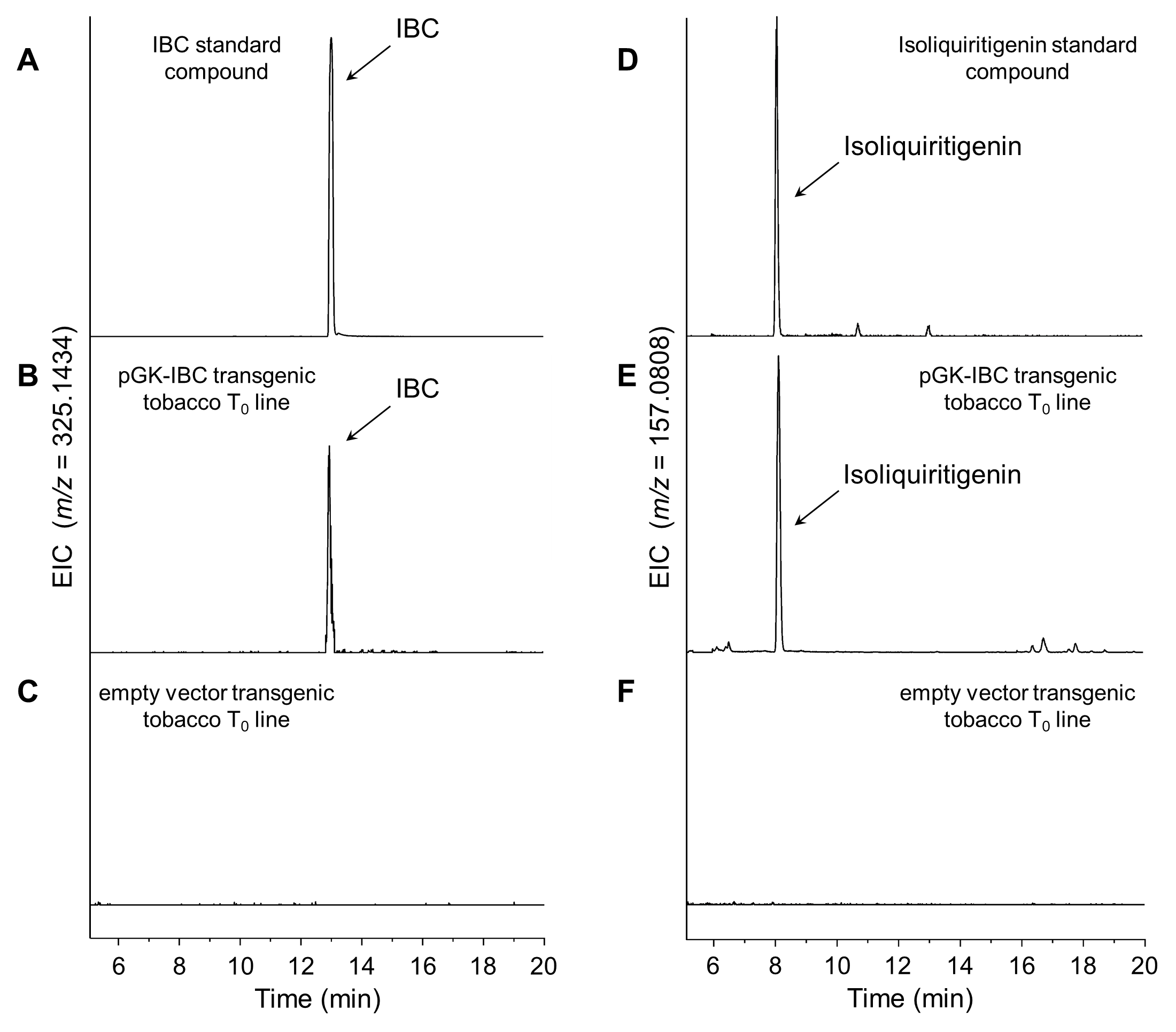


**Supplementary Figure 4.** *De novo* biosynthesis of isobavachalcone in transgenic tobacco T_0_ lines.

Representative extracted ion chromatogram (EIC) for *m/z* 325.1434 of **(A)** isobavachalcone (IBC) standard, **(B)** pGK-IBC transgenic tobacco T_0_ lines, and **(C)** empty vector transgenic tobacco T_0_ lines, and EIC for *m/z* 257.0808 of **(D)** isoliquiritigenin standard, **(E)** pGK-IBC transgenic tobacco T_0_ lines, and **(F)** empty vector transgenic tobacco T_0_ lines was shown.


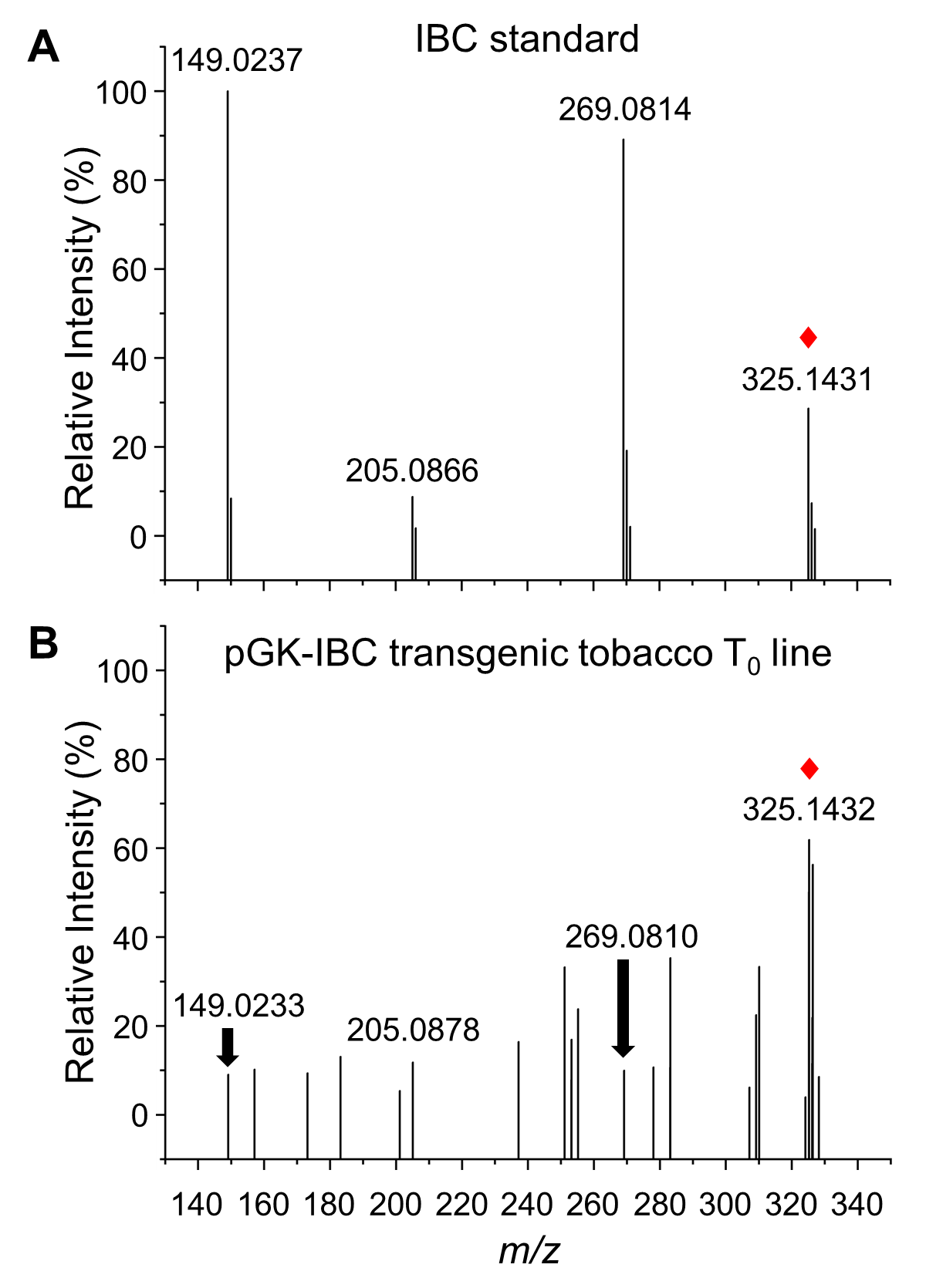


**Supplementary Figure 5.** MS/MS spectra of isobavachalcone (IBC) in **(A)** IBC standard compound sample and **(B)** pKG-IBC transgenic tobacco T_0_ lines.

## 1.2 Supplementary Tables

**Supplementary Table 1.** Primers used in this study.

| **Primer Name** | **Sequence (5' to 3')** |
| --- | --- |
| GmCHS8_Cloning_Fw | ggggacaagtttgtacaaaaaagcaggctATGGTGAGCGTAGCTGAGATCCG |
| GmCHS8_Cloning_Rv | ggggaccactttgtacaagaaagctgggtTCAGATGGCCACACTGCGCAG |
| GmCHR5_Cloning_Fw | ggggacaagtttgtacaaaaaagcaggctATGGCTGCCACCACCTTAGT |
| GmCHR5_Cloning_Rv | ggggaccactttgtacaagaaagctgggtTCATTCTTCATCCCATAGATCATCA |
| GuILDT_Cloning_Fw | ggggacaagtttgtacaaaaaagcaggctATGGATTCAATGGTTATTGGGTC |
| GuILDT_Cloning_Rv | ggggaccactttgtacaagaaagctgggtTCATCGAACTAAAGGTATGAGAAAGTA |
| SfILDT_Cloning_Fw | ggggacaagtttgtacaaaaaagcaggctATGGGTTTTGTGCTTCCTGC |
| SfILDT _Cloning_Rv | ggggaccactttgtacaagaaagctgggtTCATCTAAATAAGGGTATGAGGCAG |
| SfFPT_Cloning_Fw | ggggacaagtttgtacaaaaaagcaggctATGGGTTCTATGCTTCTTGCATC |
| SfFPT_Cloning_Rv | ggggaccactttgtacaagaaagctgggtTCATCTAAACAAAGGTATGAGGAAGTA |
| HlPT1_Cloning_Fw | ggggacaagtttgtacaaaaaagcaggctATGGAGCTCTCTTCAGTTTCTAGC |
| HlPT1_Cloning_Rv | ggggaccactttgtacaagaaagctgggtCTAAATGAACAGATATACAACGTATTC |
| AtMYB28_Cloning_Fw | ggggacaagtttgtacaaaaaagcaggct*ggtacc*ATGTCAAGAAAGCCATGTTGCGT |
| AtMYB28_Cloning_Rv | ggggaccactttgtacaagaaagctgggt*ggtacc*TCATATGAAATGCTTTTCAAGCG |
| GmCHS8_Mutation_Fw | AAAAGTTAGG***A***CTCAAACCTGAGAAGATGAAG |
| GmCHS8_Mutation_Rv | TCAGGTTTGAG***T***CCTAACTTTTGCTCAACTTGGTC |
| NtACTIN1_RT_Fw | ATGAGCAAGAGTTGGAGACTGC |
| NtACTIN1_RT_Rv | CAATGGAAGGACCAGATTCATCATATTC |
| NtACTIN1_qPCR_Fw | GAACGGGAAATTGTCCGCGA |
| NtACTIN1_qPCR_Rv | GAACCTCTCTGAGCCAATGG |
| GmCHS8_qPCR_Fw | CTCCAGACAGTGAAGGTGC |
| GmCHS8_qPCR_Rv | ATCCAAAAGATGGAGTTGTAATC |
| GmCHR5_qPCR_Fw | GCATCCAGAGATCAACTCTT |
| GmCHR5_qPCR_Rv | ATGGATCCCCACACACC |
| SfFPT_qPCR_Fw | CGGCAATCGTCAGTATTTATG |
| SfFPT_qPCR_Rv | CGGCATGTCCCAGAACC |

**Supplementary Table 2.** Final concentration of each component in MS media.

| Component | MS0 | MS1 | MS2 | MS3 |
| --- | --- | --- | --- | --- |
| Murashige & Skoog basal salt mixture | 4 g/L | 4 g/L | 4 g/L | 4 g/L |
| 2-morpholinoethanesulfonic acid hydrate | 0.5 g/L | 0.5 g/L | 0.5 g/L | 0.5 g/L |
| MS vitamin | 1 mL/L | 1 mL/L | 1 mL/L | 1 mL/L |
| Sucrose | 30 g/L | 30 g/L | 30 g/L | 30 g/L |
| Agar | 8 g/L | 8 g/L | 8 g/L | 8 g/L |
| 6-Benzylaminopurine | 0 | 1.5 mg/L | 0.2 mg/L | 0 |
| 1-Naphthylacetic acid | 0 | 0.2 mg/L | 0 | 0 |
| Indole-3-butyric acid | 0 | 0 | 0 | 0.1 mg/L |
| Cefotaxime | 0 | 400 mg/L | 200 mg/L | 0 |
| Kanamycin | 0 | 75 mg/L | 75 mg/L | 0 |
